# On the interpretation of synchronization in EEG hyperscanning studies: a cautionary note

**DOI:** 10.3389/fnhum.2013.00881

**Published:** 2013-12-24

**Authors:** Adrian P. Burgess

**Affiliations:** Aston Brain Centre, School of Life and Health Sciences, Aston UniversityBirmingham, UK

**Keywords:** electroencephalography, hyperscanning, phase synchronization, social neuroscience, inter-brain connectivity, Phase Locking Value

## Abstract

EEG Hyperscanning is a method for studying two or more individuals simultaneously with the objective of elucidating how co-variations in their neural activity (i.e., hyperconnectivity) are influenced by their behavioral and social interactions. The aim of this study was to compare the performance of different hyper-connectivity measures using (i) simulated data, where the degree of coupling could be systematically manipulated, and (ii) individually recorded human EEG combined into pseudo-pairs of participants where no hyper-connections could exist. With simulated data we found that each of the most widely used measures of hyperconnectivity were biased and detected hyper-connections where none existed. With pseudo-pairs of human data we found spurious hyper-connections that arose because there were genuine similarities between the EEG recorded from different people independently but under the same experimental conditions. Specifically, there were systematic differences between experimental conditions in terms of the rhythmicity of the EEG that were common across participants. As any imbalance between experimental conditions in terms of stimulus presentation or movement may affect the rhythmicity of the EEG, this problem could apply in many hyperscanning contexts. Furthermore, as these spurious hyper-connections reflected real similarities between the EEGs, they were not Type-1 errors that could be overcome by some appropriate statistical control. However, some measures that have not previously been used in hyperconnectivity studies, notably the circular correlation co-efficient (CCorr), were less susceptible to detecting spurious hyper-connections of this type. The reason for this advantage in performance is discussed and the use of the CCorr as an alternative measure of hyperconnectivity is advocated.

## Introduction

Over the last decade, the development of techniques that allow the measurement of neural activity from two or more individuals simultaneously, known as hyperscanning, has been heralded with some justification as a promising new field in social neuroscience (Dumas, 2011; Dumas et al., 2011; Sanger et al., 2011; Babiloni and Astolfi, 2012; Konvalinka and Roepstorff, 2012). Hyperscanning methods have been used in many different social contexts but all involve the simultaneous recording of brain activity from two or more individuals with a view to determining how co-variation in their neural activity is related to their behavioral and social interactions and this work has resulted in multiple claims that neural coupling between people is increased during social interaction. In contrast, there has been little attempt to determine how valid the methods used to measure connectivity are in this context and this paper is one attempt to redress that omission.

The first true hyperscanning study was reported by Montague et al. (2002) using two linked fMRI scanners with two individuals playing a variant of the children's guessing game, “handy-dandy.” Other studies have used near-Infrared Spectroscopy (Funane et al., 2011) and there is also a single case study demonstrating the feasibility of hyperscanning using magnetoencephalography (Baess et al., 2012). Most studies, however, have relied upon EEG which, is not only more readily available than other methods but is also better suited for use in naturalistic social settings, and these are the focus of this paper.

The first EEG hyperscanning study was reported by Babiloni et al. (2006) and involved sets of four individuals playing Tressette, a bridge-like game. Since then, there have been 30 more EEG publications that meet the definition of hyperscanning coming from more than 20 independent studies have claimed increased neural coupling between people engaged in social interaction (Babiloni et al., 2006, 2007a,b, 2011, 2012; Flexer and Makeig, 2007; Tognoli et al., 2007, 2011a,b; Chung et al., 2008; Tognoli, 2008; Yun et al., 2008; Astolfi et al., 2009, 2010a,b,c, 2011a,b, 2012; Lindenberger et al., 2009; Dumas et al., 2010, 2012a,b; Fallani et al., 2010; Dodel et al., 2011; Lachat et al., 2012; Naeem et al., 2012a,b; Sanger et al., 2012, 2013; Yun et al., 2012; Kawasaki et al., 2013). The methods used to establish neural coupling between people have been very consistent and nearly all studies have used one of three methods: (i) covariance in amplitude or power, (ii) Partial Directed Coherence (PDC); (Baccala and Sameshima, 2001), and (iii) phase synchrony, mostly the Phase-Locking Value (PLV) (Lachaux et al., 1999) or a variant thereof.

The most frequently used method for demonstrating brain-to-brain coupling between socially interacting individuals, used in 12 reports, has been to show that there are contiguous, or near contiguous changes in EEG amplitude or power (Babiloni et al., 2007b, 2011, 2012; Tognoli et al., 2007; Yun et al., 2008; Astolfi et al., 2009; Dumas et al., 2012b; Lachat et al., 2012; Naeem et al., 2012a,b; Yun et al., 2012; Kawasaki et al., 2013). In most cases, this EEG amplitude/power has been estimated from event-related changes or from FFT. Showing that there are co-variances in EEG power is a weak form of association and although it is suggestive of neural coupling, it is by no means conclusive.

The second most commonly used method has been that of PDC which was the approach used in the very first EEG hyperscanning study (Babiloni et al., 2006) and has been used in at least nine further studies since (Babiloni et al., 2007a,b; Astolfi et al., 2010a,b,c, 2011a,b, 2012; Fallani et al., 2010). PDC is based on multivariate autoregressive modeling and Granger Causality and is designed to be able to show the direction of flow of information (linear) between two systems (Baccala and Sameshima, 2001). As such, PDC seems ideally suited to role of identifying inter-brain coupling in hyperscanning studies, at least in those cases where when one person's behavior is driving another's. However, both PDC and Granger causality are not without their critics. Friston (2011), for example, provides a critique of the use of Granger causality in fMRI research and, some of the limitations he mentions apply equally well to EEG research. It is certainly the case that, as Konvalinka and Roepstorff (2012) have observed, the results of PDC in hyperscanning studies have not replicated well, but whether this is related to the use of PDC, or to some other cause, is not clear.

The final class of measures of brain-to-brain coupling all involve measures of phase synchrony (Lindenberger et al., 2009; Dumas et al., 2010, 2012a; Sanger et al., 2012, 2013; Yun et al., 2012). The first use of phase synchronization as a measure of coupling with electrophysiological data was by Tass et al. (1998), who defined synchronization as occurring when |φ_*n, m*_| < const, where const is some suitably small value, *n* and *m* are integers, φ_*n, m*_ (*t*) is the phase difference, *n*ϕ_1_ (*t*) − *m*ϕ_2_ (*t*) and ϕ_1, 2_ are the phases of the two oscillators. The most widely used index of phase locking adopted in hyperscanning studies has been the Phase Locking Value (PLV) (Lachaux et al., 1999) which is a measure that seems well suited for capturing the rapid flow of information between people in social situations. Interestingly, some hyperscanning studies have used PLV to characterize behavioral interactions even when they have used other measures of coupling for the EEG (e.g., Tognoli et al., 2007).

**Figure 1:**
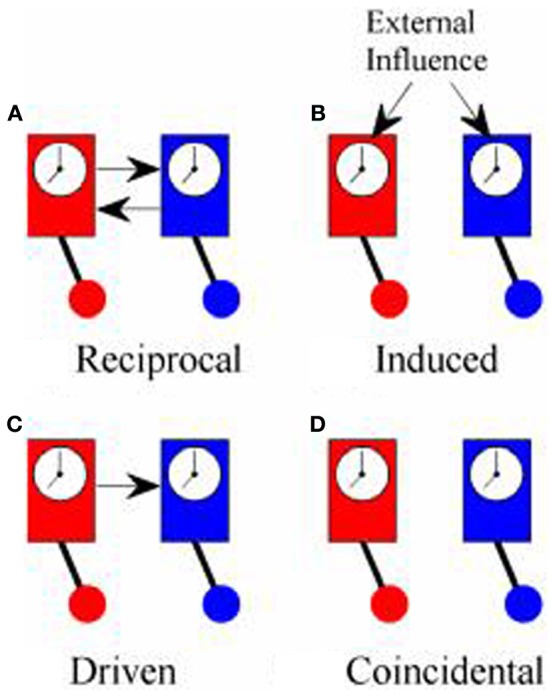
**Types of synchrony. (A)** Shows “reciprocal” synchronization whereby the pendulums of the clocks swing in phase because there is reciprocal influence between the two; **(B)** shows “induced” synchronization whereby the phase of the pendulums of both clocks are influenced by a common external driver; **(C)** shows “driven” synchronization whereby the pendulum of one clock influences the phase of the pendulum of the other clock without any reciprocal influence; **(D)** shows “coincidental” synchronization where there is no coupling between the clocks but the pendulums remain in a fixed phase relationship to each other because they both swing at the same frequency.

Although both PDC and PLV have been used to measure coupling between cortical oscillations recorded in the EEG from two or more different people, what they actually measure is quite different in each case and, for this reason, it is worth reviewing what is meant by synchronization. The first scientific description of synchronization came in 1665 from Christiaan Huygens who wrote a letter to the Royal Society in which he described “*an odd kind of sympathy*” in which the pendulums of identical clocks mounted on the same support came to swing exactly out of phase (i.e., anti-phase) regardless of the phase they had been in when they had been set running (Pikovsky et al., 2001; Klarreich, 2002). The explanation of this phenomenon is that the swing of the pendulum in one clock induced small movements in the support from which the clocks were suspended that would slightly alter the swing of the pendulum of the second clock. At the same time, the pendulum of the second clock would induce movements in the support that affected the swing of the pendulum in the first clock. These small mutual nudges would continue to shift the phase of each pendulum until they came to a point where the nudge from one would exactly counterbalance the nudge from the other and this would occur when the pendulums were precisely anti-phase. In modern terms, the two clocks were in a system of reciprocal negative feedback and would continue to change until the system reached the state of minimum energy transfer between the two. Minimum information transfer (in fact, zero energy transfer) occurs in the anti-phase condition. An example of in-phase reciprocal synchronization is shown in Figure 1A.

True synchronization then, is of interest in neuroscience because it is a reliable marker of the flow of information between elements of a system. Simply observing a consistent phase relationship between two oscillators (clocks, human brains etc.), however, does not necessarily mean that they are in the same condition of reciprocal information exchange displayed by Huygens' clocks. Synchronization might also occur if both clocks are driven by some external influence as in Figure 1B. In hyperscanning experiments, this might occur if the participants simultaneously experience the same stimuli such as watching a movie together, even though they are not directly interacting (Hasson et al., 2008). Alternatively, the influence between oscillators might be one-way with one oscillator driving another, Figure 1C, which is exactly the type of coupling that PDC is designed to identify. Each of these types of synchronization might be of interest, depending upon the context of the study, and it would often be of interest to know which type of synchronization is being observed. In practice, however, these different types of synchronization may be difficult to tell apart.

There is a fourth type of synchrony which is not really synchronization at all: coincidental synchrony, Figure 1D. This is a phenomenon which is generally of no interest and, in the context of hyperscanning, has nuisance value only. Unfortunately, it is not a rare phenomenon. Had Huygens's clocks been too far apart to influence each other, they would have remained in the same fixed phase relationship to each other indefinitely. Over time, small differences between the clocks would lead to a gradual shift in phase but, at least over short periods of time, the phase difference would be nearly constant. In general, two oscillators will show a consistent phase relationship whenever they share a common frequency of oscillation. To put this in the context of the brain, consider two adults, each with a dominant alpha rhythm of ~10 Hz sitting in isolation in separate rooms. If we were to measure their EEG, we could expect to see a fairly consistent phase relationship between their alpha rhythms, at least over short time scales, even though there is no communication between them. This situation is exactly the same as the example of the identical but unconnected pendulum clocks and stems solely from the fact that they share a common frequency of oscillation. It follows from this that simply observing a consistent phase relationship does not imply synchronization or information exchange or, as Pikovsky et al. (2001) put it, “*synchronous variation of two variables does not necessarily imply synchronization*.” The critical feature of synchronization is not that the oscillators are synchronous but that there is “… *adjustment of their rhythms, or appearance of phase locking due to interaction*” (Tass et al., 1998).

To put this more formally, two oscillators can be said to be synchronized if deviations from the regular oscillatory cycle of one oscillator provides information about deviations in the oscillatory cycle of the other. Such a definition suggests that a measure of the co-variation or correlation between oscillators might sometimes be more useful. It is for this reason, that most hyperscanning studies do not simply measure phase coupling in the EEG between individuals but compare the degree of coupling between different experimental conditions. In the best studies, the experimental conditions are identical in every way except that in one case the participants are socially engaged and in the other they are not. In practice, however, this level of experimental control is difficult to achieve.

The aim of this paper is to examine the performance of currently used measures of phase synchronization in hyperconnectivity studies (PDC and PLV) and compare them with alternative measures including coherence (COH), the circular correlation co-efficient (CCorr) and Kraskov's Mutual Information estimator (KMI) (Kraskov et al., 2004). Good performance, in this context, is defined by three qualities. First, the measure should be unbiased and have a low root mean squared error of estimation (RMSE). Specifically, when the true connectivity, *r* = 0, the estimated connectivity should be zero or very close to it. Second, the estimate of connectivity should increase monotonically as *r* increases and third, the estimate of coupling strength between two channels should be independent of the distribution of the signal in either of the constituent channels. In particular, the estimate of coupling strength should be insensitive to changes in the variance of the marginal distributions of deviations from the expected phase in either channel.

The first comparison included simulated time series where the degree of connectivity could be systematically varied. The second comparison compared EEG from individuals independently recorded but analyzed as though they had been recorded as part of a hyperscanning study. Because these EEG recordings were completely independent and there was no social contract between participants, a good measure of hyperconnectivity should not detect any synchronization between them. The first example of EEG data was from an event-related potential paradigm in which data recorded around the time of the presentation of a visual stimulus was used. This is analogous to induced synchrony (Figure 1B) as there may be some apparent connectivity between individuals because they share similar external stimulation. The second example of EEG data was from two independent resting state conditions in which there was no external stimulation to induce synchrony.

## Materials and methods

### Measures

Five different methods for estimating functional hyper-connectivity were used in this study.

#### Coherence (COH)

COH is the traditional Fourier-based method of connectivity and the Welch estimate of coherence is given by:

(1) $${\text{COH}}_{xy}=\frac{\left|\frac{1}{N}\sum_{k=1}^{N}Y_{k}\left(f\right)X_{k}^{*}\left(f\right)\right|}{\sqrt{\frac{1}{N}\sum_{k=1}^{N}X_{k}\left(f\right)X_{k}^{*}\left(f\right)}.\frac{1}{N}\sum_{k=1}^{N}Y_{k}\left(f\right)Y_{k}^{*}\left(f\right)}$$

where *X*_*i*_(ω) denotes FFT of the *k*th segment of the time series *x*(*t*) at frequency *f* and ^*^ indicates the transpose and complex conjugate. The analysis was performed using the MatLab function *mscohere.m*. COH values range from 0 to + 1.

#### Partial directed coherence (PDC)

The PDC from *y* to *x* is defined as:

(2) $${\text{PDC}}_{xy}\left(f\right)=\frac{A_{xy}\text{​}\left(f\right)}{\sqrt{a_{y}^{*}\text{​}\left(f\right).a_{x}\text{​}\left(f\right)}}$$

where *A*_*xy*_(*f*) is an element in ***A***(*f*) which is the Fourier Transform of the multivariate autoregressive (MVAR) model coefficients, ***A***(*t*), of the time series ; *a_y_*(*f*) is *y*th column of ***A***(*f*). MVAR and PDC analysis was performed using the Extended Multivariate Autoregressive Modeling Toolbox for MatLab (Faes and Nollo, 2011). PDC values range from 0 to + 1 but as PDC_*x, y*_ ≠ PDC_*y, x*_ both are reported.

#### Phase locking value (PLV)

There is an unfortunate terminological confusion over the use of the term “PLV” as, not only is it often referred to as the Phase Locking Index (PLI) but, both “PLV” and “PLI” can refer to two quite different measures that have equations of the identical form but quite different meaning. The PLV, as originally defined by Lachaux et al. (1999), is estimated by:

(3) $${\text{PLV}}_{n}=\frac{1}{N}\left|\sum_{k=1}^{N}e^{i\left(\phi_{\left(t,\;k\right)}-\psi_{\left(t,\;k\right)}\right)}\right|$$

where *N* is the number of trials, ϕ_(*t, n*)_, is the phase on trial, *n* at time *t*, in channel ϕ and ψ_(*t, n*)_ in channel ψ. The PLV_*n*_ varies between 0 and 1 where 1 indicates perfect phase locking and 0 indicates no phase locking. This form of the PLV_*n*_ is a measure of the consistency of the phase difference and is related to the inter-trial variance of the phase difference, σ^2^_ϕ − ψ_, by the relationship PLV_*n*_ = 1 − σ^2^_ϕ−ψ_. Because this form of the PLV_*n*_ is based on the phase difference across trials, it is only suitable for event-related paradigms.

However, there is a variant of the Equation (3) that has been frequently used in EEG hyperscanning studies which involves averaging the instantaneous phase differences over time within a single trial:

(4) $${\text{PLV}}_{t}=\frac{1}{T}\left|\sum_{n=1}^{T}e^{i\left(\phi_{\left(t,\;n\right)}-\psi_{\left(t,\;n\right)}\right)}\right|$$

where *T* is the number of time points. This form of the PLV is essentially a measure of the intra-trial consistency of the phase difference between channels. As will become clear, this small difference between Equations (3) and (4) has important implications for the interpretation of EEG hyperscanning methods. In an attempt to remove any ambiguity, we shall refer to the measure defined by Equation (3) as the trial-averaged PLV or PLV_*n*_ and that described by Equation (4) as the time-averaged PLV, PLV_*t*_. PLV values range from 0 to + 1.

The PLV is a measure of the consistency of the phase-difference but, as noted above, simply observing that there is a consistent phase relationship between two signals does not imply covariance or information exchange or between them. Indeed, the PLV_*t*_ cannot distinguish between coincidental phase synchronization and true phase synchronization. To see why phase difference is a poor measure of information exchange, consider the variance of the difference in the case of the bivariate normal distribution^1^ which is given by:

(5) $$\sigma_{x-y}^{2}=\sigma_{x}^{2}+\sigma_{y}^{2}-2\sigma_{x}\sigma_{y}\rho ,\;$$

where σ^2^ is the variance and ρ is the correlation between the two distributions *x* and *y*. Clearly σ^2^_*x* − *y*_ can be small, indicating strong association between the two variables, not only when ρ is large but when σ^2^_*x*_ and σ^2^_*y*_ are small. This means that although σ^2^_*x* − *y*_ is related to ρ, it is a rather poor proxy for it and makes no sense to measure correlation this way in such cases. The natural measure of correlation in this case is the Pearson Product Moment Correlation Coefficient which measures the covariance of the deviations from the expected (i.e., mean) values of the two variables.

#### Circular correlation coefficient (CCorr)

The Pearson Product Moment Correlation Coefficient is not suitable for use with circular distributions like phase but there are several suitable candidates including the Circular Correlation Coefficient (CCorr) (Jammalamadaka and Sengupta, 2001), CCorr is a direct parallel to the Pearson Product Moment Correlation Coefficient for circular data and is given by:

(6) $${\text{CCorr}}_{\phi ,\psi }=\frac{\sum_{k=1}^{N}\sin\text{​}\left(\phi -\bar{\phi }\right)\sin\text{​}\left(\psi -\bar{\psi }\right)}{\sqrt{\sum_{k=1}^{N}\sin^{2}\text{​}\left(\phi -\bar{\phi }\right)\sin^{2}\text{​}\left(\psi -\bar{\psi }\right)}}$$

where ϕ and ψ are the mean directions for channels 1 and 2 respectively. For oscillatory signals, like the EEG, phase is approximately uniformly distributed and the population mean directions are not defined. However, in the case of uniform marginal distributions, any arbitrary direction can be defined as the mean without ill effect although for convenience, the sample mean directions, ϕ and ψ were always used. Unlike PLV_*t*_, the Circular correlation, CCorr, is much more robust to coincidental synchronization. The reason for this is that CCorr measures the circular covariance of differences between the observed phase and the expected (i.e., mean) phase. In the case of a perfect oscillator, the frequency of oscillation will be constant and there will be no variance. In the case of a sinusoidal oscillation, knowing the frequency of oscillation and its phase at any single time point provides a complete description of its behavior. For imperfect oscillators, as all real-world oscillators are, there will be small variations in phase over time. However, knowing the phase of such an oscillator in its recent past makes it possible to predict its phase in the near future. In the case of two related channels, if one channel is slightly in advance of its expected phase at a given time, then the phase in the other channel is also likely to be advanced (for positively correlated signals; the reverse for negatively correlated signals). That is, the phase variance of the oscillators co-varies and this is what CCorr measures. In the case of two unrelated channels, the phase variance will not co-vary and the CCorr will be zero. As the PLV measures the phase difference, which is a poor proxy for phase covariance, it is likely to be poorer at discriminating between related and unrelated signals. CCorr was measured using the CircStat toolbox for MatLab (Berens, 2009). CCorr values range from 0 to + 1.

#### Kraskov mutual information (KMI)

The KMI is a non-parametric estimator of mutual information (Kraskov et al., 2004) based nearest-neighbor method for estimating entropy proposed by Kozachenko and Leonenko (1987) cited in Beirlant et al. (1997). The KMI, adapted for use with phase data, is given by:

(7) $$I_{\phi \psi }=\Psi \text{​}\left(k\right)+\Psi \text{​}\left(N\right)-\sum_{i=1}^{N}\left(\Psi \text{​}\left(n_{\phi \left(i\right)}+1\right)+\Psi \text{​}\left(n_{\psi \left(i\right)}+1\right)\right)\;\;\;\;$$

where Ψ(.) is the digamma function, *n*_ϕ (*i*)_ is the number of points with ‖ϕ_*i*_ − ϕ_*j*_‖ ≤ ε (*i*)/2 and *n*_Ψ (*i*)_ is the number of points with $\left\|\psi_{i}-\psi_{j}\right\|\leq \frac{\varepsilon \left(i\right)}{2}$; ε(*i*) is the distance from observation *i* to its *j*^th^ nearest neighbor and distances are measured with respect to the maximum norm ε (*i*) = max {ε_ϕ_ (*i*), ϕ_ψ_ (*i*)} and *N* is the total number of independent observations. In the simulations reported here, *j* = 5 and the distances were angular distances.

For convenience, all mutual information values were transformed to the range 0–1 using the relationship $r=\sqrt{1-e^{-2I_{\phi \psi }}}$ where, *I*_ϕΨ_, is the mutual information between ϕ and Ψ and, *r*, is the correlation from a bivariate normal distribution with the same mutual information.

### Simulations

**Figure 2:**
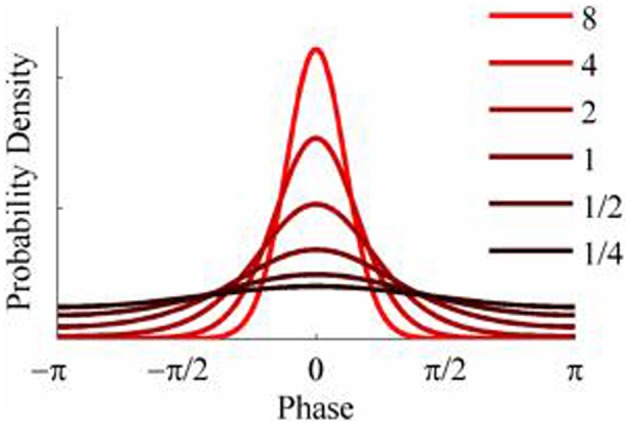
**Probability density functions of the von Mises distribution for different values of concentration, κ**.

**Figure 3:**
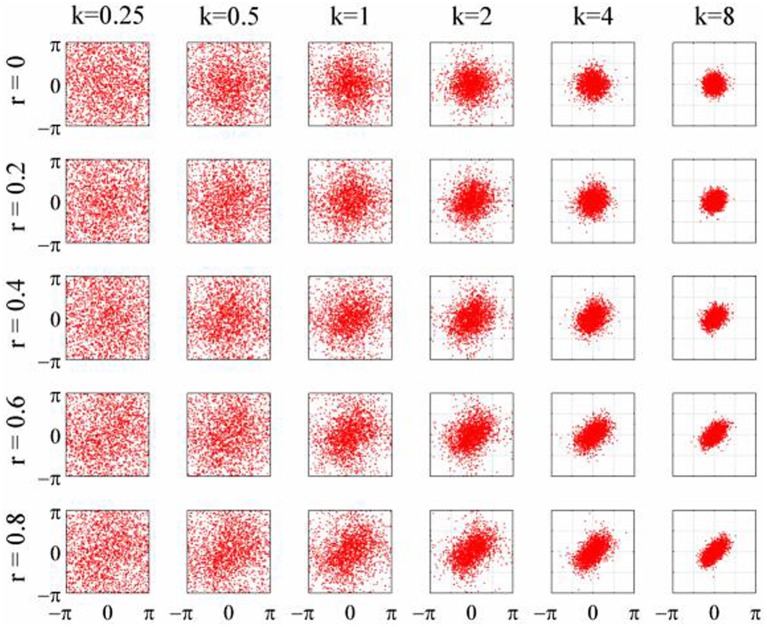
**Scatterplots of the 2D-von Mises distribution for different values of concentration, κ, and correlation, *r***.

The objective of the simulations was to generate time series when the phase-coupling between the two could be systematically varied. Phase distributions can be generated from the von Mises distribution, a circular analog of the Gaussian distribution that ranges from -π to + π. The von Mises distribution is defined by its mean direction, μ, and concentration, κ, which are analogous to the Gaussian mean, μ, and the reciprocal of the variance,1/σ^2^, respectively. Examples of the von Mises distribution for μ = 0 and varying values of κ are shown in Figure 2. The von Mises distribution can be generalized to two dimensions where phase can be represented as a distribution on the surface of a torus (Singh et al., 2002). The covariance between the two dimensions of the bivariate von Mises distribution is controlled by a parameter λ. The joint probability density function is defined by the 5 parameters (μ_1_, μ_2_, κ_1_, κ_2_, and λ) and from this it is a simple matter of numerical integration to calculate the mutual information between the two distributions (see Appendix). Given the probability density function of a 2-D von Mises distributions it is a simple matter to generate random variables with any different levels of mutual information and concentration (Figure 3) using the acceptance/rejection method (Gentle, 1998).

**Figure 4:**
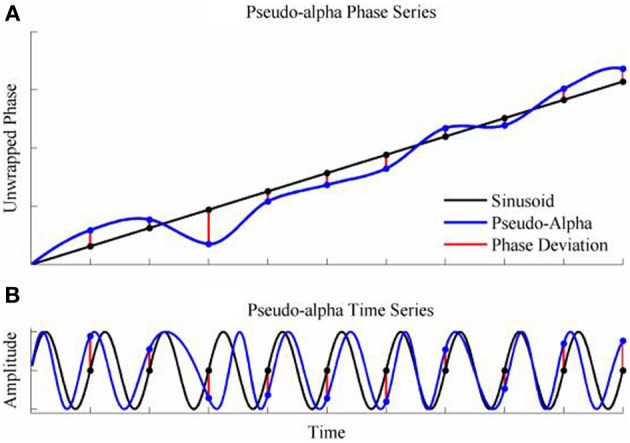
**Simulation of the pseudo-alpha rhythm. (A)** Shows the unwrapped phase of a regular 10 Hz sinusoid (Black line) with random phase deviations generated from the von Mises distribution (Red lines) and added to the sinusoid at 100 ms interval. The phase series for the pseudo-alpha rhythm is formed by a smooth line (Blue line) that connects the points of the sinusoid + phase deviation. **(B)** Shows the times series generated by taking the sine of the phase series in **(A)**.

To generate a time series with randomly varying phase shifts, we first generated an unwrapped and perfectly regular phase series [0, 2π, 3π …. *n*π ], and generated *n* independent samples from a von Mises random distribution [ϕ_1_; ϕ_2_; ϕ_3_;…. ϕ_*n*_] and added the two together giving a new phase series [0 + ϕ_1_, 2π + ϕ_2_, 3π + ϕ_3_,….*n*π + ϕ_*n*_]. The von Mises random variables were added as phase deviations to the expected regular phase series. In this case, the aim was to simulate an alpha rhythm with a mean frequency of *f* = 10 Hz sampled at = 500 Hz. The phase series [0, 2π, 3π ….*n*π] corresponded to a time series of [0, 0.1, 0.2, 0.3… *n*/*f*]s so the phase values for intermediate time points from 0 to *n*/*f* seconds in 1/ϖ second intervals were estimated by spline interpolation. Finally, the pseudo-alpha rhythm was obtained by taking the sine of the interpolated phase series. This created a smoothly frequency-varying oscillation with constant amplitude in which the variance of the frequency was determined by, κ, the concentration parameter of the von Mises distribution. In these simulations, therefore, 1/κ is a measure of the variance of the marginal distributions of deviations from the expected phase. An example is shown in Figure 4. It is a simple matter to generalize this process to the 2D cases using random variables a 2D-von Mises Distribution and the degree of dependency can be controlled by the parameter λ. For the simulations values of λ were chosen to approximate bivariate correlations of [0, 0.2, 0.4, 0.6, 0.8] and the concentration values of, κ, were [0.25, 0.5, 1, 2, 4, 8]. One hundred samples of 100 s epochs of pseudo-alpha were generated for analysis for each value of λ and κ.

In order to generate pseudo-alpha time series in which there was a time-lagged dependency between channels, *n* + 1 independent samples were drawn from a 2D-von Mises distribution [ϕ_1_; ϕ_2)_; ϕ_3_;…. ϕ_*n* + 1_] and added to the phase series giving two new phase series [0 + *f*_(1, 1)_, 2π + ϕ_(2, 1)_, 3π + ϕ_(3, 1)_…. *n*π + ϕ_(*n*, 1)_] and [0 + ϕ_(2, 2)_, 2π + ϕ_(3, 2)_, 3π + ϕ_(4, 2)_,….*n*π + ϕ_(*n* + 1, 2)_]. The rest of the procedure was identical to that for the zero-lagged time series but with the result that the two pseudo-alpha time series were maximally correlated with a lag of 100 ms but uncorrelated at lag 0. That is, one time series “caused” the other in the Granger sense.

#### Hyperconnectivity analysis

COH was estimated for each pair of time series using Welch's method with non-overlapping Hamming Windows of 1024 ms Equation (1). For PDC, an MVAR model was generated for each 100 s pair of time series using a model order determined by the Akaike Information Criterion. The PDC was estimated from the MVAR coefficients following Equation (2). COH and PDC values were averaged across each of the 100 randomizations.

Estimates of PLV_*t*_, CCorr, and KMI were derived from the instantaneous phase of the time series. Instantaneous phase at each time point in each time series was estimated from the Hilbert Transform of the pseudo-alpha data using FFT with a window of 1024 ms and it was these estimates that were used for estimating coupling strength. The Hilbert Transform produces a “real” and “imaginary” time series and the phase was estimated by $\phi \left(t\right)=\tan^{-1}\left(\frac{\text{Imag}\left(t\right)}{\text{Real}\left(t\right)}\right)$. In all cases, the Hilbert-estimated phases were very close to “true” phase values that had been entered into the simulation. The phase-series were divided into epochs of 1024 ms and the PLV_*t*_ and CCorr were estimated for each using Equations (4) and (6) respectively. The resulting values were averaged across all epochs and all randomizations. This procedure of estimating hyperconnectivity over short epochs and averaging follows the methods reported in the literature (Lindenberger et al., 2009; Dumas et al., 2010, 2012a; Sanger et al., 2012, 2013; Yun et al., 2012) each of whom used segments of EEG of less than 800 ms.

As estimation of KMI assumes independent observations, the instantaneous phase data was down-sampled to a rate equal to the mean frequency of the signal i.e., 10 Hz. An estimate of KMI was derived for each of the down-sampled segments of pseudo-alpha phase data using Equation (7) and averaged across the 100 random samples.

#### Statistical analysis

Each of the measures of connectivity was evaluated in terms of their bias and Root Mean Squared Error of Estimation (RMSE) for the case where the true connectivity, *r*, was zero. Bias and RMSE were defined as: $\text{Bias}=\frac{1}{N}\sum_{k=1}^{N}{(r}_{i}-{\hat{r}}_{i})$ and $\text{RMSE}=\frac{1}{N}\sqrt{\sum_{k=1}^{N}{\left(r_{i}-{\hat{r}}_{i}\right)}^{2}}$ where *r_i_* is the true connectivity and ${\hat{r}}_{i}$ is the estimate of the true connectivity. Note that for COH, PDC and PLV_*t*_, where the values are defined to be greater than or equal to zero, the Bias and RMSE are equal. Mutual information, by definition must also be greater than or equal to zero but, the KMI estimator can produce small negative values and for this reason, Bias will not always be equal to RMSE.

## Human EEG

### Participants

The data used for this study are a subset of a dataset that has previously been reported on and full details of the experiment are reported in Burgess (2012). Participants were 10 healthy young adults (5 women, 5 men) recruited through advertisement with a mean age of 25.4 years (*SD* = 5.8; range 20–40). Written informed consent was obtained from all subjects and the experiment was conducted as approved by the Riverside Research Ethics Committee. All investigations were conducted according to the principles expressed in the Declaration of Helsinki and data were analyzed anonymously.

#### Procedure

EEG was recorded from participants at rest (60 s Eyes Open Relaxed and 60 s Eyes Closed Relaxed) and as they were presented with a series of faces. There were 90 trials which included the presentation of a fixation cross for 1000 ms followed by a photograph of a face for the same duration. Each photograph was of the head and shoulders of a man or woman with neutral emotional expressions, facing directly toward the participant. The inter-trial interval consisted of a blank screen and randomly varied between 1000 and 2000 ms. All data were recorded from participants completely independently and at separate times. There was no social interaction between any of the participants at any time during the recording of these data.

#### Materials and equipment

Twenty-eight electrodes were positioned on the scalp using an ECI electrode cap with electrodes placed according to the International 10–20 system with an additional nine electrodes: Oz, FC5/6, CP1/2, CP5/6 PO1/2. Horizontal electro-oculogram (EOG) was recorded from the external canthus of each eye, and the vertical EOG was recorded from the supra- to suborbit of the left eye. Electrode impedances were all under 5 kΩ. EEG and EOG were amplified using a 32 channel Neuroscan Synapse-II System. Signal bandpass was 0.1–100 Hz and the digital sampling frequency was 500 Hz. Reference was to the left ear and converted to average reference offline.

#### Data preparation

For the resting state, data were divided into consecutive epochs of 1024 ms. For the event-related paradigm, EEG was divided into pre-stimulus and post-stimulus epochs each of 1024 ms duration. The pre-stimulus epochs included data from −1024 ms to −1 ms and the post-stimulus epochs contained data from + 1 to + 1024 ms where zero was defined as the time of stimulus onset.

For both data sets, epochs including values outside the range −100 to + 100 μV range were excluded from the analysis. In order to facilitate the comparison between EEG recorded from different individuals, it was convenient to ensure that each participant contributed the same amount of data. For this reason, only the first 20 epochs for the resting state conditions and the first 50 epochs for the event-related paradigm were included.

#### Hyperconnectivity analysis

The data from each participant was paired with every other participant and analyzed as if they had been recorded jointly in a hyperscanning experiment. With 10 participants, this gave a total of 45 pseudo-pairings, one of whom was arbitrarily nominated as participant 1 and the other as participant 2. Twenty-eight channels of EEG were recorded for each person meaning that there were 56 channels for each pair of participants giving a total of 1540 possible different channel combinations. Of these, only the 784 hyper-connections that paired data between people were considered further.

For each pairing, EEG data were concatenated across epochs in preparation for the hyperconnectivity analysis. For the resting state data, 20 consecutive epochs of artifact-free EEG were joined together from each condition to form 20.48 s of data for each of the eyes open and eyes closed conditions. For the event-related data, 50 epochs of pre-stimulus and post-stimulus EEG were concatenated separately to give two time series of 51.2 s each.

COH and PDC were estimated from these concatenated data for each participant separately using the method described for simulated data and hyperconnectivity estimates were the highest values obtained in each of the Theta (4–8 Hz), Alpha (8–12), Beta1 (13–19 Hz), Beta2 (20–29 Hz), and Gamma (30–70 Hz) frequency bands. For the phase-based measures, PLV_*t*_, CCorr and KMI, the concatenated data were filtered into the same frequency bands using Butterworth filters and the instantaneous phase was estimated using the Hilbert transform in the same way as for the simulated data. PLV_*t*_ and CCorr were estimated for each 1024 ms epoch and frequency band separately and averaged. The KMI was estimated from the same data down-sampled to 10 Hz.

#### Statistical analysis

For the rest conditions, connectivity in the Eyes Open and Eyes Closed conditions were compared and for the event-related data, connectivity in the pre-stimulus period was compared to that in the post-stimulus period. The difference in connectivity between experimental conditions was estimated for each of the 784 electrode pairs and averaged across each of the 45 pseudo-pairs of participants.

In order to determine if the differences were reliable, a randomization testing procedure was used to control the Type-1 error (Holmes et al., 1996; Burgess and Gruzelier, 1997). Consider one electrode pair; under the null hypothesis, there should be no difference between conditions and so randomly swapping the data between them and calculating the difference many times should provide a good estimate of the variability in the connectivity of that electrode pair that is due to chance. If the difference in connectivity observed in the real data set is larger than 95% of the differences observed in the randomized data sets, it is reasonable to say that that difference is greater than might be expected by chance. To extend this idea to multiple electrode pairs, instead of examining the distribution of the connectivity difference at each electrode pair in turn, the distribution of the largest difference in connectivity across all electrode pairs for each randomization was examined. The 95th percentile of the distribution of the maximum difference represents the value that would not be exceeded at any electrode pair by chance. In this way, the family-wise Type-1 error can be controlled to 5%.

The maximum difference across all 784 electrode pairs was estimated for 1000 randomizations of the data. The 95th percentile of this distribution was used as the upper cut-off for determining statistical significance and controlling the per-condition comparison Type-1 error to 5%. The same process was used to obtain a lower cut-off value.

## Results

### Simulations

**Figure 5:**
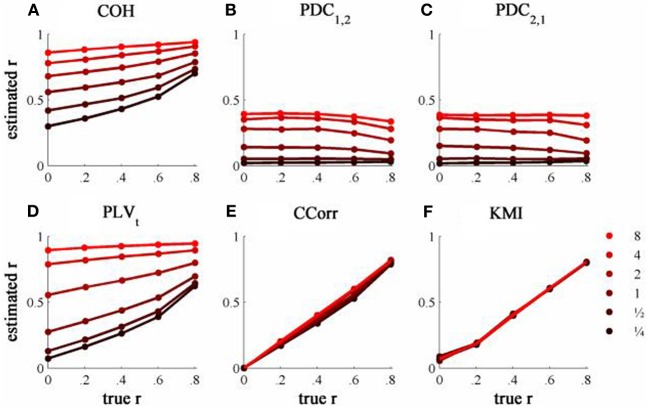
**The relationship between the true and estimated coupling for each measure of connectivity for the zero-lagged simulated data at different levels of concentration, κ. (A)** Shows coherence, **(B,C)** partial directed coherence, **(D)** time-averaged phase-locking value, **(E)** circular correlation coefficient and **(F)** Kraskov mutual information. Concentration values are ¼, ½, 1, 2, 4, and 8.

**Figure 6:**
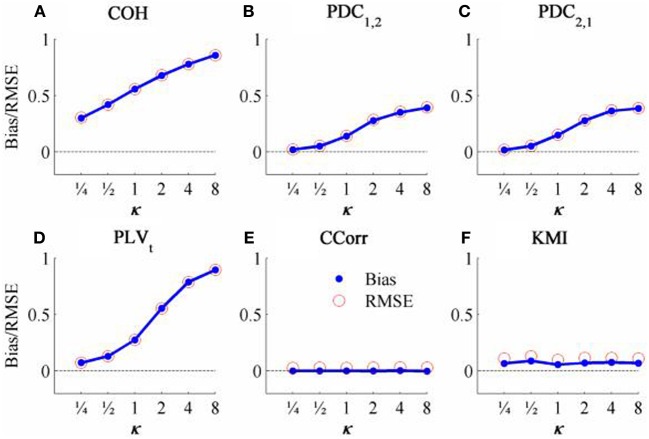
**The bias and RMSE for each connectivity measure estimated from the zero-lagged simulated data at different levels of concentration, κ, when the coupling, *r* = 0**. Blue dots indicate bias and red circles RMSE. **(A)** Shows coherence, **(B,C)** partial directed coherence, **(D)** time-averaged phase-locking value, **(E)** circular correlation coefficient and **(F)** Kraskov mutual information. For COH, PDC and PLV, as all values are >0, bias = RMSE are equal. For CCorr and KMI, where values may be ≤ 0, bias ≠ RMSE.

The results from the simulations showing the effects of varying the concentration, κ, and the zero-lagged correlation, *r*, on each of the measures of connectivity are shown in Figure 5. The first criterion of good performance, that the measures should have low bias and low RMSE can be addressed by examining the mean bias and RMSE of each of the connectivity measures when *r* = 0 and for each value of κ (Figure 6). Note that for COH, PDC and PLV, the bias equals the RMSE as all values are positive and greater than 0; only for CCorr and KMI do they differ. COH did not meet the criterion for any value of *r* and PDC and PLV_*t*_ only came close for low values of κ. KMI was close to the criteria for all values of κ but, as the minimum value of KMI is zero, there was a small, consistent bias. Only CCorr met the criteria fully.

The second and third criteria of good performance, that the estimate of connectivity should increase monotonically as *r* increases and that it should be insensitive to changes in the variance of the marginal distributions of deviations from the expected phase (1/κ), can be considered together. Table 1 shows the proportion of variance in each measure of connectivity that can be accounted for by r, κ, the interaction *r* by κ and error derived from a Two-Way ANOVA of the simulation data. For all of the measures, except PDC, *r*, accounted for a good proportion of the variance but for COH and PLV, this proportion was small compared to the proportion attributable to κ. The poor performance of PDC is this context was unsurprising as it is designed to identify Granger causality in which one time series leads the other, not instantaneous associations as seen here. Nevertheless, the sensitivity of PDC to κ meant that relatively high values of PDC were obtained even where there was no real association between channels and it was the measure that showed the highest proportion of error variance. The interaction, *r* by κ, was important only for the PLV_*t*_ where it accounted for 7.5% of the variance and was manifest as a relatively greater influence of *r*, at low values of κ (Figure 5C). The two measures that best met the criteria were CCorr and KMI as they were both overwhelmingly influenced by *r* but not κ and, of the two, CCorr had a much smaller error variance.

**Table 1:** **Showing the proportion of variance accounted in each connectivity measure by correlation and concentration**.

**Connectivity measure**	**Zero-lagged data source of variance (%)**				**100 ms-lagged data source of variance (%)**			
	***R***	**κ**	***r* by κ**	***Error***	***R***	**κ**	***r* by κ**	***Error***
COH	18.2	77.3	2.5	2.0	16.7	78.4	2.8	2.1
PDC_1, 2_	1.2	85.8	1.0	12.0	39.5	56.9	0.9	2.7
PDC_2, 1_	0.4	86.4	0.9	12.3	22.1	55.3	16.1	6.6
PLV_*t*_	15.6	76.2	7.9	0.3	0.8	97.8	1.1	0.2
CCorr	99.0	0.3	0.2	0.6	62.7	2.1	1.4	33.8
KMI	95.6	0.0	0.0	4.4	0.0	0.1	0.0	99.9

**Figure 7:**
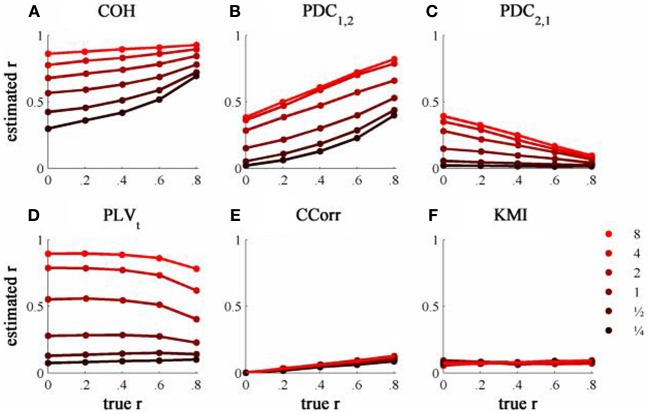
**The relationship between the true and estimated coupling for each measure of connectivity for the 100ms-lagged simulated data at different levels of concentration, κ. (A)** Shows coherence, **(B,C)** partial directed coherence, **(D)** time-averaged phase-locking value, **(E)** circular correlation coefficient and **(F)** Kraskov mutual information. Concentration values were ¼, ½, 1, 2, 4, and 8.

The results from the simulations showing the effects of varying the concentration, κ, and the 100 ms-lagged correlation, *r*, on each of the measures of connectivity are shown in Figure 7. This simulation was designed to provide an example of Granger Causality that would be well-suited for analysis by PDC. The first point to note is that COH was largely unaffected by the change (compare Figures 5A and 7A) and performed badly with both sets of data. In contrast, the PLV, CCorr, and KMI were all adversely affected which is unsurprising as these measures are not designed for use in this context. In the case of PLV_*t*_ and KMI, less than 1% of the variance was attributable to *r*. For PLV_*t*_ most variance was accounted for by κ whereas for KMI it was error. The poor performance of KMI with this data set occurred because it was estimated from the phase-series down-sampled to 10 Hz, the same rate at which the random phase deviations were added to the phase sequence. This meant that the simultaneous estimates of phase were truly independent. In contrast, because the PLV_*t*_ and CCorr were estimated based on intermediate points that were spline estimates of the preceding and subsequent phase deviations, each datum contained some information about the lagged relationship between the phase series. This is the reason why CCorr shows some sensitivity to increases in *r*, although much less than for the zero-lagged data. Of course, each of these measures would perform much better if they had been estimated across a range of time lags.

As expected, PDC performed better on this simulation than with the zero-lagged data. PDC_1, 2_ showed a clear monotonic increase with *r* correctly showing that channel 1 led channel 2. Similarly, PDC_2, 1_ showed a monotonic decrease with *r*, meaning that the predictability of channel 1 given channel 2 diminished as the predictability of channel 2 increased. However, in both cases, the largest proportion of variance was attributable to κ, not *r*.

### Human studies

#### Event-related changes in synchrony

**Figure 8:**
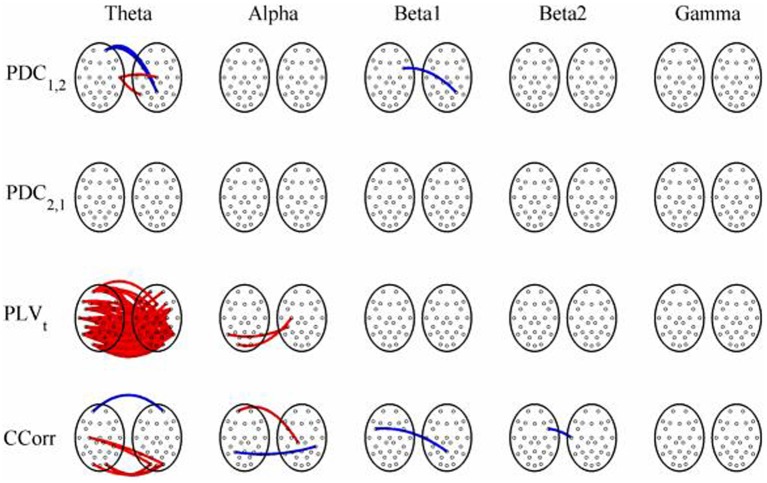
**Significant changes in mean time-averaged hyperconnectivity between pre- and post-stimulus conditions by connectivity measure and frequency band**. The rows represent the hyperconnectivity results for each of the measures used (PDC_1, 2_, PDC_2, 1_, PLV_*t*_ and CCorr) and the columns represent the frequency bands (theta, alpha, beta1, beta2, and gamma). The pairs of large circles in each cell represent the heads of the participants in a pseudo-pair. The smaller circles indicate the topographical location of the EEG recording electrodes. For PLV_*t*_ and CCorr, lines drawn between the heads joining electrode sites indicate that there was a significant change in connectivity from the pre- to the post-stimulus periods between the first member of a pseudo-pair and the second member. Red lines indicate an increase in connectivity from the pre- to the post-stimulus period and blue lines indicate a decrease. For PDC_1, 2_, lines connecting electrode sites between the heads show that neural activity in the first member of a pseudo pair was more predictive of the neural activity of the second member of the pair in the post-stimulus period than in the pre-stimulus period. Allocation to first or second member of the pseudo-pair was arbitrary.

**Figure 9:**
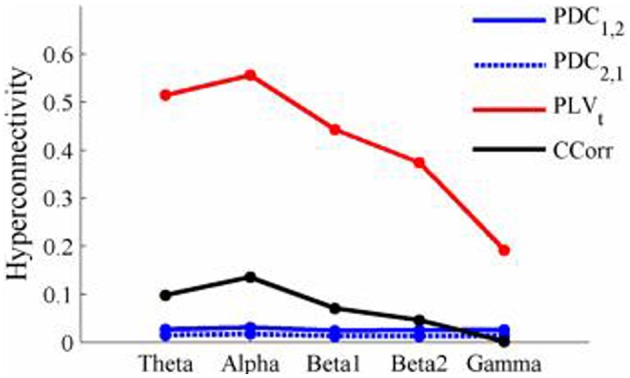
**Mean Hyperconnectivity values for time-averaged event-related EEG by connectivity measure and frequency band**.

The results of the hyperconnectivity analysis between pre-stimulus and post-stimulus conditions, controlled for multiple comparisons, are shown in Figure 8. As all the data were recorded independently, there can have been no true synchronization between the recordings. Nevertheless, there were a small number of significant changes in synchronization between the pre- and post-stimulus conditions identified by PDC and CCorr and a very large number for the PLV_*t*_. For PDC and CCorr, the changes in synchronizations involved both increases and decreases but for PLV_*t*_, they were exclusively in the direction of increased synchrony in the post-stimulus period. The estimates of mean synchrony averaged across the pre- and post-stimulus periods for each of the connectivity measures are shown in Figure 9. For PDC, the estimated levels of synchrony were consistently very low (range 0.01–0.03) and were also low for CCorr but more variable (range 0.001–0.14). In contrast, the mean synchronization was much higher for PLV with values ranging from 0.19 to 0.56.

**Figure 10:**
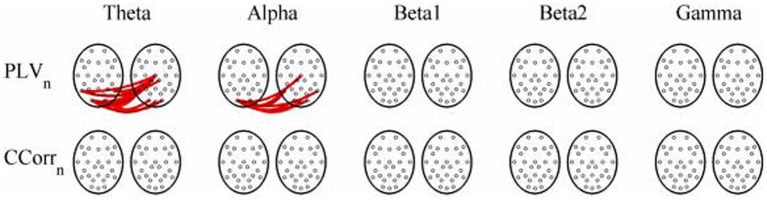
**Significant changes in mean trial-averaged hyperconnectivity between pre- and post-stimulus conditions by connectivity measure and frequency band**. The rows represent the hyperconnectivity results for each of the measures used (PLV_*n*_ and CCorr_*n*_) and the columns represent the frequency bands (theta, alpha, beta1, beta2, and gamma). The pairs of large circles in each cell represent the heads of the participants in a pseudo-pair. The smaller circles indicate the topographical location of the EEG recording electrodes. Lines drawn between the heads joining electrode sites indicate that there was a significant change in connectivity from the pre- to the post-stimulus periods between the first member of a pseudo-pair and the second member. Red lines indicate an increase in connectivity from the pre- to the post-stimulus period and blue lines indicate a decrease.

**Figure 11:**
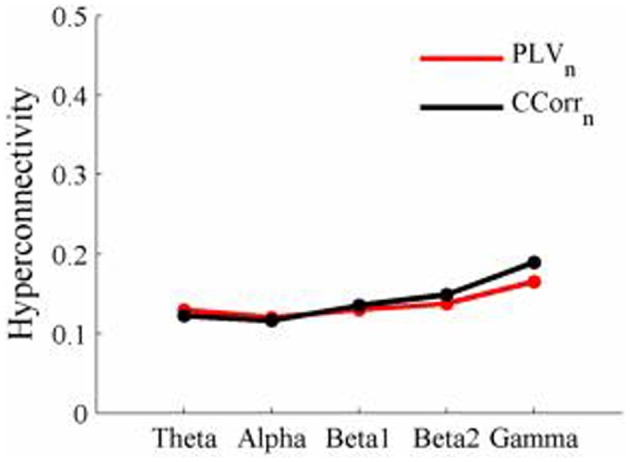
**Mean Hyperconnectivity values for trial-averaged event-related EEG by connectivity measure and frequency band**.

As these data were from an event-related paradigm, it was also possible to estimate the between-trial synchronization using PLV_*n*_ and CCorr_*n*_. Figure 10 shows the significant differences in PLV_*n*_ and CCorr_*n*_ between the pre- and post-stimulus periods. There were no significant differences in synchronization between conditions using CCorr_*n*_ but there were several using PLV_*n*_ in the theta and alpha frequency ranges. The estimates of mean synchrony in the pre- and post-stimulus periods for PLV_*n*_ and CCorr_*n*_ are shown in Figure 11. The PLV_*n*_ and CCorr_*n*_ were rather larger than their time-averaged equivalents and were approximately equal across the frequency bands (PLV_*n*_ range 0.12–0.17; CCorr range 0.12–0.19).

#### Resting state

**Figure 12:**
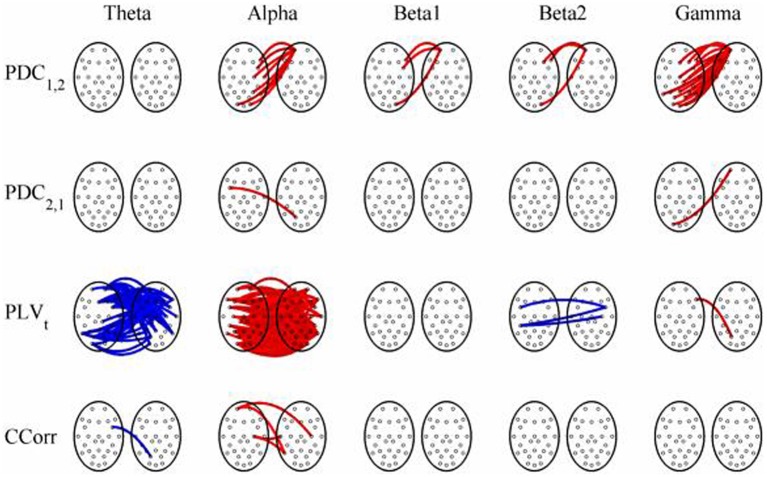
**Significant changes in mean time-averaged hyperconnectivity between eyes open and eyes closed resting states by connectivity measure and frequency band**. The rows represent the hyperconnectivity results for each of the measures used (PDC_1, 2_, PDC_2, 1_, PLV_*t*_, and CCorr) and the columns represent the frequency bands (theta, alpha, beta1, beta2, and gamma). The pairs of large circles in each cell represent the heads of the participants in a pseudo-pair. The smaller circles indicate the topographical location of the EEG recording electrodes. For PLV_*t*_ and CCorr, lines drawn between the heads joining electrode sites indicate that there was a significant change in connectivity from the pre- to the post-stimulus periods between the first member of a pseudo-pair and the second member. Red lines indicate an increase in connectivity from the pre- to the post-stimulus period and blue lines indicate a decrease. For PDC_1, 2_, lines connecting electrode sites between the heads show that neural activity in the first member of a pseudo pair was more predictive of the neural activity of the second member of the pair in the post-stimulus period than in the pre-stimulus period. Allocation to first or second member of the pseudo-pair was arbitrary.

**Figure 13:**
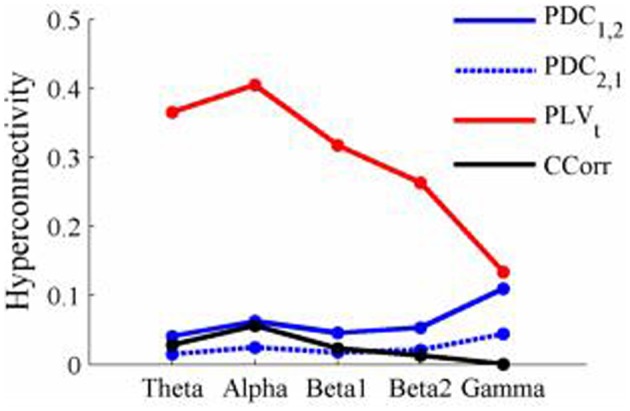
**Mean Hyperconnectivity values for time-averaged resting state EEG by connectivity measure and frequency band**.

The results of the hyperconnectivity analysis between the eyes open and eyes closed resting states, controlled for multiple comparisons, are shown in Figure 12. There were a number of significant differences in hyperconnectivity between eyes open and eyes closed using PDC. Most of these indicated that neural activity at multiple sites in pseudo-pair participant 1 was a significantly stronger predictor of neural activity at electrode site FP1 in participant 2 when the eyes were closed than when they were open. There were also two links indicating/that neural activity participant 2 drove neural activity in participant 1. There were also a small number of hyper-connections identified by CCorr, one showing significantly lower synchronization between the participants in the eyes closed condition in theta frequency range and four showing the reverse in the alpha frequency range. However, by far the largest numbers of significant changes in synchrony were identified by PLV_*t*_. In the theta frequency range, there were multiple hyper-connections that were significantly higher in the eyes open condition than in the eyes closed condition. In the alpha frequency range, there was an even larger number of hyper-connections that were greater in the eyes closed condition. The estimates of mean synchrony in the eyes open and eyes closed conditions for each of the connectivity measures are shown in Figure 13 As was the case with the event-related data, mean connectivity was low for PDC (range 0.01–0.11) and CCorr (0.001–0.06) but was very much greater for PLV_*t*_ (range 0.13–0.40).

## Discussion

The issue of how best to measure hyperconnectivity depends in no small part on what one is trying to measure. Many hyperconnectivity researchers intended to measure synchronization which, in the Huygens sense means that two oscillators (in this case, the EEG of two people) interact in such a way that their cycles become synchronous. However, synchronization, as defined by the PLV, is rather different and simply means there is a consistent phase difference between the two signals but does not necessarily imply covariance between them. By this criterion, any pair of EEG channels with a common dominant frequency would be synchronized, which surely makes this definition too inclusive to be useful. Instead, a more useful definition is that two oscillators can be said to be synchronized if deviations from the regular oscillatory cycle of one oscillator provides information about deviations in the oscillatory cycle of the other.

By this definition, none of the commonly used measures of connectivity fared well in the simulations. COH, PDC, and PLV were biased measures of the co-variation between phase series and, under a broad range of conditions provided inaccurate estimates of the true hyperconnectivity. In particular, they were each prone to detect hyperconnectivity that didn't exist. It is well known that COH is a biased estimator of true coherence (Maris et al., 2007) but using Welch's method limits the extent of the problem. PLV too, is a biased estimator of coupling strength and the bias is greater when small samples of data are used, particularly, as is the case with PLV_*t*_, when non-independent data points are used (Vinck et al., 2012). To put the scale of the problem in context, consider those simulations where the concentration was close to the mean value seen in the human EEG recordings and the true hyperconnectivity was zero (κ = 2, *r* = 0). Here the estimated coupling strengths were 0.65, 0.19, and 0.58 for COH, PDC, and PLV respectively.

These spurious couplings are not solely due to the familiar bias of the estimators. Rather, the coupling was driven by changes in the variances of the individual phase series (i.e., 1/κ of the marginal distributions of deviations from the expected phase). As Table 1 shows, COH, PDC, and PLV_*t*_ were more sensitive to changes in the variance of the marginal distributions of deviations from the expected phase than to changes in the covariance of the phases (Table 1). The result is that any change in the variance of the marginal distributions of deviations from the expected phase will be identified as a change in hyperconnectivity whether or not there is any real change in the covariance of the signals. Indeed, using PLV to measure hyperconnectivity is akin to trying to determine the correlation between two continuous variables by measuring the variance of the difference between them; the difference is related to co-variance (see Equation 5), but only indirectly so.

Instead, it may be more appropriate to use a measure that estimates the co-variation of the distributions directly. Both COH and PDC measure the co-variation between the signals (to be precise, the cross-power spectral density) and so should be suitable for this purpose. However, both methods assume that the covariance between signals is stationary throughout an epoch, which in our simulations, it was not. The rapidly changing phase shifts in our simulations are the most likely reason for the poor performance of COH and PDC here. CCorr also estimates the co-variation of the distributions directly but does not assume a constant phase relationship across each epoch and we were able to show in the simulations that it provides an unbiased estimate of hyperconnectivity with a very low RMSE. In addition, we showed that a more general measure of hyperconnectivity, which estimates mutual information rather than phase-covariance, KMI, also performs well, although there was a small positive bias in the estimates and the computational demands were much greater.

The persuasiveness of simulations depends in no small measure on how realistic one perceives them to be, so it is often helpful to supplement them with evidence from real data. By creating pseudo-pairs of participants from EEG data recorded in completely independent sessions, and analyzing them as if their data had been collected during a hyperscanning study, we could be confident that any hyper-connections observed would be spurious. We considered two conditions. The first was an event-related paradigm that might be expected to generate spurious hyper-connections because the participants were subject to similar sensory experiences and this comparison was designed to emulate the case of induced synchrony (Figure 1B). The second was a comparison of two resting states (eyes open and eyes closed) in which there was no exogenous stimulation and this comparison was designed to emulate the case of co-incidental synchrony (Figure 1D).

In both the event-related and resting-state paradigms, PDC and CCorr each identified a small number of spurious hyper-connections that differed between conditions. Most of these connections were weak (< 0.1) and some showed an increase in hyperconnectivity whilst others showed a decrease and they can easily be dismissed as Type-1 errors. The only exception to this was the anomalous finding of multiple spurious hyper-connections using PDC_1, 2_ focused on a single electrode (FP1).

A very different pattern was seen in the case of PLV_*t*_, however. In the event-related data, nearly 20% of all possible connections in the theta frequency band (*n* = 145) were erroneously found to be significantly higher in the post-stimulus period. In addition, the strength of connectivity was strong with a mean PLV_*t*_ of 0.51. There were also multiple spurious hyper-connections found using the trial-averaged PLV_*n*_ with 14 (1.8%) and 10 (1.3%) found in the theta and alpha frequency bands although the strength of the connections was weak, 0.13 and 0.12 respectively. In the resting state comparisons, PLV_*t*_ showed a decrease in hyper-connectivity from the eyes open to the eyes closed conditions in 54 cases (7%) whilst in alpha, there was a corresponding increase in 170 (22%) hyper-connections and again, the strengths of the connections were moderately strong with a mean value of 0.37 in theta and 0.41 in alpha.

This strong and systematic pattern of findings using PLV in these very different paradigms is troubling because, in the absence our knowledge that these hyper-connections must be spurious, they might easily have been accepted as real. Such a large number of hyper-connections cannot easily be dismissed as Type-1 errors. The problem of multiple comparisons is well-understood by hyperconnectivity researchers and most recent studies have included appropriate statistical mechanisms to control the family-wise Type-1 errors that would otherwise ensure. In this case, a robust and well-established method for controlling the family-wise Type-1 error control had been used but the real problem is that spurious connections were found despite these precautions. The clear implication is that statistical control of Type-1 errors is not sufficient to guard against detecting spurious connections.

Far from being a statistical artifact, it is likely that the large numbers of spurious hyper-connections identified by PLV_*t*_ arose from real similarities between the EEG recorded from different participants. In general, any systematic difference between the experimental conditions that affects the variance of the phase difference of the EEG recorded, will affect the PLV. This might occur in a number of ways but would include, for example, a systematic difference in rhythmicity between conditions. Any strong oscillatory component in the EEG means that the phase at any time point is much more predictable (i.e., the phase variance is lower). If the phase variance in one or both EEG channels is reduced, the variance of the phase difference will also be reduced and this means that PLV_*t*_ will be higher.

For this to happen, it is necessary that the change in rhythmicity is one that reliably occurs in most individuals but this is not difficult to achieve. The remarkably consistent, yet reliably predictable responses of the EEG to challenges attest to this (e.g., event-related potentials and event-related desynchronization). Given the same stimulation and cognitive and motor demands, any arbitrarily chosen group of neurotypical participants will produce event-related changes in their EEG that look very much like those produced by any other neurotypical group. Change the stimuli or the demands, and the topography and time-frequency characteristics of the responses will change in predictable ways. In short, different people presented with the same conditions will produce similar EEG responses.

Most of our spurious hyper-connections can be explained through this mechanism. Consider the resting state comparisons. The difference between the eyes open and eyes closed resting states is typically characterized in terms of the Berger effect in which opening the eyes severely attenuates the alpha rhythm. That is, the rhythmicity in alpha is greater when the eyes are closed than when they are open. We should expect, therefore, that in the alpha frequency band, PLV_*t*_ would be higher when the eyes were closed and this is what we observed. In addition, there is a stronger theta rhythm in the eyes open condition than in the eyes closed condition so we should expect to find higher PLV_*t*_ with eyes open, and this too was seen (Figure 12).

The same phenomenon can account for the spurious hyper-connections seen with the event-related data. The presentation of a visual stimulus, like a face, will induce a power increase in the theta frequency range in the post stimulus period (i.e., theta synchronization) (Burgess and Gruzelier, 1997). The presence of a stronger oscillatory component in the post-stimulus period meant that phase variance was lower than in the pre-stimulus period giving higher PLV_*t*_ values (Figure 8). One might also have expected a reduction in PLV_*t*_ in alpha because the presentation of a visual stimulus is invariably followed by a power reduction in that frequency range (alpha desynchronization) but this was not seen in this case.

A similar mechanism can account for the spurious synchronizations detected by the trial-averaged PLV_*n*_. The presentation of a visual stimulus induces a phase-re-organization of the ongoing EEG (Burgess, 2012). In the pre-stimulus period, a cross-section across trials at any given time point, would show that the phases were randomly distributed. In the post-stimulus period, although the EEG is not strictly phase-locked, the phase-variance is much reduced and this reduction of phase-variance within each channel means that the phase difference between channels will also be reduced. The result is the increase in PLV_*n*_ that we observed (Figure 10).

The important point to note is that the statistically significant but spurious differences in PLV observed derived not from any connection between the participants involved but from the fact that our experimental conditions were associated with systematic differences in the rhythmicity of the EEG. This has two important implications for the field of hyperscanning. First, it means that spurious hyper-connections are likely to be found under a broad range of experimental conditions as any systematic difference between conditions in terms of movement, stimulus presentation or mentation could have this effect. Second, these spurious connections are not Type-1 errors that can be overcome using a statistical control for multiple comparisons.

There are two obvious ways to tackle this problem: improved experimental control and the use of a different measure of phase synchronization. There is certainly no substitute for good experimental design and if the conditions to be compared can be matched in terms of stimulus presentation and movement, and if appropriate control conditions are used, then much of this problem would be resolved. Indeed, this is already the case with the better designed studies in the field. However, although it might be possible to obtain this level of experimental control in restricted social situations, one of the key attractions of hyperscanning is that it has the potential to open a window on the neural co-ordination of people socially interacting in the real world. Not for the first time, strict experimental control and ecological validity stand in opposition to one another.

The other approach to tackle this problem is to adopt an alternative measure of phase synchronization. Any measure that is sensitive to changes in the marginal distributions of deviations from the expected phase is also likely to be sensitive to changes in the rhythmicity of the EEG. Although PLV was the most problematic measure in this context, at least in terms of detecting spurious hyper-connections in human EEG, the simulations showed that PDC and COH were also vulnerable in this respect, at least under certain circumstances. The real problem is that, although the PLV is widely used as a measure of phase synchronization, a high value of PLV does not necessarily mean there is any true phase synchronization at all. If we wish to claim that two time series, or, in this case, two phase series, are related to each other, we need to show that deviations from the dominant frequency in one oscillator co-vary with deviations in the other. Had the pendulums on Huygens's clocks simply shown a consistent phase relationship to each other, he would never have discovered the phenomenon of phase synchronization. What surprised him was not that the pendulums remained in the same fixed phase relationship to each other where they'd started, but that they progressively shifted phase until their swings became aligned. As Pikovsky et al. (2001) put it, “*This adjustment of rhythms due to interaction is the essence of synchronization*.”

This emphasis on synchronization has been unfortunate because what most EEG hyperscanning researchers wish to show is that cortical oscillations from different people are systematically related to each other in a way that depends upon their social interactions. This means that we need to show that there is covariance (or more generally, mutual information) between the EEG of the people concerned. Synchronization is one way of doing this but, as this study has shown, there may be advantages from using a measure of correlation instead. Fortunately, we have at least two candidate measures that might serve: CCorr and KMI. CCorr is insensitive to changes in the marginal distributions of deviations from the expected phase and, hence, resistant to changes in the rhythmicity of the EEG because it measures the co-variation between phase series. Adopting this measure, or some suitable alternative such as KMI, may not solve the problem completely, but it may go a long way to reducing the risk of detecting spurious hyper-connections in future.

To conclude, existing measures of hyper-connectivity are biased and prone to detect coupling where none exists. In particular, spurious hyper-connections are likely to be found whenever any difference between experimental conditions induces systematic changes in the rhythmicity of the EEG. These spurious hyper-connections are not Type-1 errors and cannot be controlled statistically. Measures of the co-variance or mutual information between phases-series provide more robust evidence of true hyperconnectivity and are to be preferred in this context.

## Author contributions

Adrian P. Burgess designed the study, supervised the data collection, performed all the analysis and simulations and wrote the paper and sang the theme tune.

### Conflict of interest statement

The author declares that the research was conducted in the absence of any commercial or financial relationships that could be construed as a potential conflict of interest.

## Footnotes

^1^Unfortunately, the equivalent equation for the bivariate von Mises distribution is not known.

## Appendix: the 2 dimensional von moses distribution

The probability density function of the von Mises distribution in given by:

(A1) $$f\text{​}\left(\phi \right)=\frac{e^{\kappa \cos\left(\phi -\mu \right)}}{2\pi I_{0}\text{​}\left(\kappa \right)}$$

where ϕ is the phase (−π < ϕ < π), μ and 1/κ are analogous to the mean and variance of the normal distribution respectively and *I*_0_(κ) is the zero-order modified Bessel function. In two dimensions, circular variables can be represented as a probability distribution on a torus and a convenient parallel to the bivariate normal distribution is given by the two dimensional von Mises distribution whose probability density function is given by Singh et al. (2002):

(A2) $$\begin{array}{l} f\text{​}\left(\phi ,\psi \right)=\frac{1}{\text{C}}e^{[\kappa_{\phi }\cos\left[\left(\phi -\mu_{\phi }\right)\right]\;+\;\kappa_{\psi }\cos\left[\left(\phi -\mu_{\psi }\right)\right]}\\ \;{\;}^{+\;\lambda \sin\left[\left(\phi -\mu_{\phi }\right)\right]\sin\left[\left(\psi -\mu_{\psi }\right)\right]]} \end{array}$$

where C is a normalizing constant and λ is a parameter describing the statistical dependency between the two distributions ϕ and ψ. Concentration values of, κ, were defined so that κ_ϕ_ = κ_ψ_ and the values used in the simulations were [0.25, 0.5, 1, 2, 4, 8]—see Figure 2. The mutual information, *I*_ϕψ_, between distributions depended upon λ and κ and could be estimated accurately through numerical integration (Hnizdo et al., 2008). Values of λ were selected for each value of κ, that generated distributions with mutual information values of [0, 0.0204, 0.0872, 0.2231, 0.5108]. For convenience, mutual information values were converted to putative correlation values by the relationship $r=\sqrt{1-e^{-2I_{\phi \psi }}}$ giving values of [0, 0.2, 0.4, 0.6, 0.8] respectively.
